# Regioisomer-independent quantification of fatty acid oxidation products by HPLC-ESI-MS/MS analysis of sodium adducts

**DOI:** 10.1038/s41598-019-47693-5

**Published:** 2019-08-01

**Authors:** Katelyn W. Ahern, Vlad Serbulea, Catherine L. Wingrove, Zachary T. Palas, Norbert Leitinger, Thurl E. Harris

**Affiliations:** Department of Pharmacology, University of Virginia Health System, Charlottesville, VA 22908 United States

**Keywords:** Lipid peroxides, Lipidomics

## Abstract

Despite growing acknowledgement of the role of oxidized fatty acids (oxFA) as cellular signaling molecules and in the pathogenesis of disease, developing methods to measure these species in biological samples has proven challenging. Here we describe a novel method utilizing HPLC-ESI-MS/MS to identify and quantify multiple full-length oxFA species in a regioisomer-independent manner without the need for time-consuming sample preparation or derivatization. Building on recent progress in the characterization of FA and their oxidation products by MS/MS, we employed positive-ion ionization by measuring sodium adducts in conjunction with Differential Energy Qualifier Ion Monitoring to unequivocally verify the presence of the hydroperoxide, hydroxide, and ketone oxidation products of linoleic and arachidonic acid. Our HPLC method achieved separation of these oxidized species from their unoxidized counterparts while maintaining regioisomer-independent elution, allowing quantification over a 5 log_10_ range with a lower limit of quantification of 0.1 picomoles. With a simple sample preparation and a runtime as low as 11 minutes, our method allows the rapid and facile detection and measurement of full-length oxFA in biological samples. We believe this approach will allow for new insight and further investigation into the role of oxFA in metabolic disease.

## Introduction

Impaired redox homeostasis has been implicated in a wide array of pathologies, including obesity, stress-induced insulin resistance, atherosclerosis, rheumatoid arthritis, and hypertension^1–4^. One hallmark of oxidative stress in these pathologies is increased lipid oxidation^5,6^. While oxidized lipids can be produced enzymatically in a tightly regulated fashion by lipoxygenases and cyclooxygenases to form signaling molecules, such as leukotrienes and prostaglandins^7,8^, non-enzymatic lipid oxidation during oxidative stress may surpass cellular control and lead to destructive biological processes, such as membrane damage, protein modification, DNA oxidation, deposition of atherogenic plaque, and tissue inflammation^9–12^. Due to the wide-ranging implications, there has been much interest in examining the diverse species of the oxidized lipidome and their differential biological functions.

Of note, recent work by our group has demonstrated relative changes in the components of this oxidized lipidome, specifically increases in full-length oxidized phospholipids, to be associated with the onset of high-fat diet-induced obesity leading to dramatic differences in adipose tissue macrophage polarization and tissue inflammation^12^. These effects on macrophages were found to be entirely dependent on the oxidized fatty acid (oxFA). Remarkably, treatment with non-esterified oxidized arachidonic acid (oxAA) alone was able to comparably induce antioxidant, metabolic, and proinflammatory gene expression^13^. As a result, a means of specifically identifying and quantifying these full-length oxFA in biological samples is of great importance.

Due to rapid expansion of the lipidomics field, analytical methods have been compiled by various groups such as LIPID MAPS. High-performance liquid chromatography-mass spectrometry (HPLC-MS) is gaining in popularity as the preferred way to identify and quantify various lipid classes; the major exception is fatty acids (FA), which have been traditionally measured by gas chromatography-mass spectrometry (GC-MS) following derivatization^14,15^. While GC methods measuring FA have high sensitivity and resolution, they have inherent problems and require extensive sample preparation, making them unsuitable for measuring oxFA. GC requires high temperatures to produce volatile analytes for detection that would be destructive to oxFA, which are thermolabile^7^. To improve volatilization and prevent breakdown, oxFA would require multiple derivatization steps^16^. Not only are these steps time-consuming, but they inherently prevent the distinction of various oxFA species, such as hydroperoxides and hydroxides. Without derivatization, FA are measured by MS using negative ion formation due to the loss of the acidic hydrogen of the carboxylic acid which is enhanced under basic conditions. However, HPLC methods used in conjunction with MS for FA measurement usually rely on reverse phase chromatography which requires the presence of a weak acid to keep the carboxylic acid in its protonated state and allow retention on the column for separation. As such, formation of the carboxylate anion in the electrospray droplets is usually suppressed^17–19^. With reactive oxFA naturally present at much lower levels than their unoxidized counterparts, this loss of sensitivity has further hindered their measurement. As such, much work and many technological improvements have been dedicated to developing means of measuring FA and their oxidation products by HPLC-MS.

Recent progress towards utilizing HPLC-MS to measure FA oxidation has primarily focused on identifying specific regioisomers either through an untargeted approach, which only has relative quantitation capability, or through a targeted approach, of which some methods allow absolute quantitation^7,16,20–25^. Meanwhile, methods focused on measuring these species irrespective of regioisomer rely predominantly on HPLC with chemiluminescence detection, which, while easily quantitated, is inherently limited in its specificity^8,26,27^. These methods measure oxFA solely based on the oxidized moiety and are unable to distinguish between individual FA backbones^8,26,27^. A method that could quantitate specific oxFA in a regioisomer-independent manner would be highly biologically relevant, as it is likely that the sum total oxidation products, rather than the formation of one particular isomer, is more important in pathologies implicating chronic oxidative stress^12,28^. Thus, our goal was to utilize HPLC coupled with electrospray ionization tandem mass spectrometry (ESI-MS/MS) to develop a targeted, species-specific method using positive-ion ionization that could identify and quantify multiple full-length oxFA species in a regioisomer-independent manner without relying on time-consuming sample preparation, such as derivatization.

## Materials and Methods

### Chemicals and reagents

All solvents used throughout were HPLC-MS grade from Fisher Scientific (Hampton, NH). Butylhydroxytoluene (BHT) used during extraction and HPLC-grade sodium acetate used during HPLC-MS/MS analysis were obtained from Sigma Aldrich (Darmstadt, Germany). Standards for linoleic acid (LA); arachidonic acid (AA); heptadecanoic acid (C17:0); ±13-hydroperoxy-9Z,11E-octadecadienoic acid (HPODE); ±13-hydroxy-9Z,11E-octadecadienoic acid (HODE); 9-oxo-10E,12Z-octadecadienoic acid (KODE); ±12-hydroperoxy-5Z,8Z,10E,14Z-eicosatetraenoic acid (HPETE); ±5-hydroxy-6E,8Z,11Z,14Z-eicosatetraenoic acid (HETE); and 5-oxo-6E,8Z,11Z,14Z-eicosatetraenoic acid (KETE) were purchased from Cayman Chemical (Ann Arbor, MI). All FA standards were used without further purification.

3T3-L1 preadipocytes were acquired from Zen-Bio (Research Triangle Park, NC). High glucose DMEM (with and without phenol red), 100x antibiotic-antimycotic, and newborn calf serum were obtained from Gibco (Hampton, NH). Fetal bovine serum was purchased from Gemini Bio-Products (West Sacramento, CA). The isobutylmethylxanthine and dexamethasone used for differentiation were from Sigma Aldrich (Darmstadt, Germany). Humulin R regular recombinant human insulin was procured from Eli Lilly (Indianapolis, IN).

### Fatty acid oxidation

OxFA mixtures were prepared by auto-oxidation of either LA or AA following a protocol adapted from phospholipid auto-oxidation^29^. From stock solutions in methanol, 1 mg of LA or AA was transferred to a glass test tube and dried down under a stream of nitrogen. The tube was loosely covered with foil to deter the introduction of contaminants and exposed to atmospheric oxygen for up to 96 h. The progression of oxidation was assessed by both direct infusion-MS/MS and HPLC-MS/MS analysis, as described below.

### ESI-MS/MS conditions

Analysis was performed on an AB Sciex 4000 QTRAP hybrid triple quadrupole-linear ion trap mass spectrometer with a Turbo V source with positive-ion ionization (Washington, D.C.). Instrument operation and data acquisition/analysis were performed using AB Sciex 1.6.3 Analyst software (Washington, D.C.). General source parameters were set as follows: ion spray voltage (IS) to 5.5 kV, interface temperature (TEM) to 180 °C, curtain gas (CUR) to 30 psi, collision activated dissociation gas (nitrogen, CAD) to 4 psi, nebulizer gas (GS1) to 25 psi, and auxiliary gas (GS2) to 10 psi. Species-dependent MS/MS parameters, including declustering potential (DP), collision energy (CE), and collision exit potential (CXP), were optimized using direct infusion-MS/MS analysis of purchased standards (Table 1).

**Table 1:** Optimized MS/MS conditions for oxFA detection and quantification.

Fatty Acid Species	Molecular Ion	Molecular Ion *m/z*	DP (eV)	Quantitative Transition	Quantitative Transition CE (eV)	Qualitative Transition	Qualitative Transition CE (eV)	CXP (eV)	QIR	QIR Range (75–125%)
LA	[M + Na]^+^	303.3	46	303.3 → 303.3	10	303.3 → 303.3	16	14	2.09	1.56–2.61
AA	[M + Na]^+^	327.3	36	327.3 → 327.3	13	327.3 → 327.3	17	6	1.97	1.47–2.46
HPODE	[M + Na]^+^	335.3	70	335.3 → 335.3	10	335.3 → 335.3	19	14	1.96	1.47–2.45
HODE	[M + Na]^+^	319.3	76	319.3 → 319.3	13	319.3 → 319.3	18	8	1.96	1.47–2.45
KODE	[M + Na]^+^	317.3	81	317.3 → 317.3	13	317.3 → 317.3	22	10	1.91	1.43–2.39
HPETE	[M + Na]^+^	359.3	46	359.3 → 359.3	7	359.3 → 359.3	14	14	1.72	1.29–2.15
HETE	[M + Na]^+^	343.3	56	343.3 → 343.3	13	343.3 → 343.3	16	10	2.18	1.63–2.73
KETE	[M + Na]^+^	341.3	71	341.3 → 341.3	13	341.3 → 341.3	20	12	1.94	1.45–2.43
C17:0	[M + Na]^+^	293.5	51	293.5 → 293.5	7	293.5 → 293.5	13	10	1.68	1.26–2.10

MS/MS conditions were optimized for detection of the single sodium adduct of each analyte; specifically declustering potential (DP), quantitative transition collision energy (CE), qualitative transition CE, and collision exit potential (CXP). Values are listed in electrovolts (eV). The listed qualifier ion ratio (QIR) is based on the noted pseudo-molecular transitions measured using the listed optimized MS/MS conditions in combination with the HPLC method outlined in the text. Based on the intensity of the qualitative transition chosen and accepted standards for QIR variation (75–125%), the tolerable range in QIR for each species was calculated.

Direct infusion-MS/MS analysis was performed on dilutions of 10 μM for individual standards or 100 μM for oxFA mixtures prepared in methanol supplemented with 200 μM sodium acetate. Samples were continuously infused with a syringe pump at a rate of 20 μL/min. Optimized MS/MS conditions (Table 1) for improved detection were determined using the Compound Optimization tool in the Analyst software as well as confirmation of detection and fragmentation in manual tuning mode at a range of DP and CE. All mass spectra were recorded over a *m/z* range of 50–500 in multi-channel analysis (MCA) mode under optimized conditions. Full-scan mass spectra depict 238 cumulative scans of 0.5 s. Product ion scans depict 79 cumulative scans of 1.5 s.

For oxFA quantitation, HPLC-MS/MS analysis in multiple reaction monitoring (MRM) mode was utilized. Using the Differential Energy Qualifier Ion Monitoring (DiffE QIM) approach of species identification, two pseudo-molecular MRM transitions, where both the parent ion and product ion mass were set to the molecular ion adduct, were monitored for each analyte: the quantitative transition had a low CE and the qualitative transition had a high CE yielding approximately 50% intensity^30^. Species identification was performed by comparing the ratio of the quantitative and qualitative transitions under the specified conditions. The qualifier ion ratio (QIR) must be in the range of 75–125% of the pure reference standard when the qualitative transition has an intensity of 50% of the quantitative transition. Further deviation indicates the likely presence of a confounding coeluting species^30–36^. Quantification was achieved by comparison to five-point calibration curves ranging from 0.1 to 1000 pmol/injection (Supplementary Fig. S1). For each species, the lower limit of quantification (LOQ) was determined to be the concentration at which the lower intensity, qualitative transition has a signal-to-noise ratio of greater than 3:1 (Table 2). Because this method relies on DiffE QIM, the limit of detection (LOD) and LOQ are the same, as the oxFA cannot be identified without the qualitative transition.

**Table 2:** Method validation parameters.

Fatty Acid Species	Intra-assay Precision (%)	Interassay Precision (%)	Accuracy (%)	LOQ (pmol)	Retention Time (min)	Relative Retention Time
LA	0.8	5.2	96–109	0.01	10.89 ± 0.019	0.95
AA	1.7	6.9	88–104	0.01	10.85 ± 0.022	0.95
HPODE	3.7	6.0	82–101	0.1	10.50 ± 0.011	0.90
HODE	1.2	8.0	94–104	0.1	10.31 ± 0.031	0.90
KODE	6.5	10.0	98–110	0.1	10.27 ± 0.028	0.90
HPETE	2.5	8.2	81–100	0.1	10.37 ± 0.023	0.91
HETE	2.0	4.3	91–111	0.1	10.47 ± 0.027	0.92
KETE	6.2	10.8	98–113	0.1	10.42 ± 0.025	0.91
C17:0	1.7	8.0	95–102	10	11.11 ± 0.055	1.00

The intra-assay precision was determined by measuring five concentrations three times each on the same day. The interassay precision was determined by measuring five concentrations on five days. Precision values are expressed as the percent value of the standard deviation compared to the mean value. Accuracy was determined by measuring three different, known concentrations five times. Accuracy values are expressed as the percent value of the calculated concentration compared to the known concentration. The lower limit of quantification (LOQ) was determined to be the concentration at which the qualitative transition had a signal-to-noise ratio of greater than 3:1. The variation in retention time was determined by comparing retention times on five days and is expressed as the average retention time and standard deviation. The relative retention time compared to the internal standard C17:0 was calculated for each species. This value is constant for all three described HPLC methods.

### HPLC conditions

A Shimadzu LC-20AD HPLC system (Kyoto, Japan) was used in combination with a DGU-20A3 degasser, SIL-20ACHT autosampler, and a CTO-20A column oven. The autosampler was maintained at 4 °C and set to an injection volume of 10 μL. Figures depict analysis performed with a Supelco Discovery C18 column (5 μm, 2.1 × 50 mm; Darmstadt, Germany) in combination with a Supelguard Discovery C18 guard column (5 μm, 2.1 × 20 mm; Darmstadt, Germany). Selective elution was achieved with a gradient of Solvent A (80:20 water:methanol) and Solvent B (methanol containing 200 μM sodium acetate) with an overall flow rate of 300 μL/min. The column was first equilibrated for 5 min with 100% Solvent A. After that, Solvent B was increased linearly to 100% over 4 min before being held constant for 4 min. Solvent A was then increased to 100% over 1 min and held constant for 3 min to rinse residual salt from the HPLC-MS/MS setup. Retention time variation was calculated for each oxFA species by comparing the retention times on five separate days (Table 2). The HPLC was connected to a Sciex 4000 QTRAP operated using the settings indicated previously.

An alternate, compatible HPLC method for a shorter runtime utilizing the same column and HPLC setup is as follows. The column was first equilibrated for 2 min with 100% Solvent A. After that, Solvent B was increased linearly to 100% over 1 min before being held constant for 4 min. Solvent A was then increased to 100% over 1 min and held constant for 3 min to rinse residual salt. An alternate, compatible HPLC method for increased separation utilized the same HPLC setup but with a Thermo Fisher Acclaim 120 C18 column (5 µm, 4.6 × 100 mm). In this method, the column was first equilibrated for 2 min with 100% Solvent A. After that, Solvent B was increased linearly to 100% over 1 min before being held constant for 12 min. Solvent A was then increased to 100% over 1 min and held constant for 5 min to rinse residual salt (Supplementary Fig. S2).

### Validation procedure

For determination of method precision, five concentrations (0.1 to 1000 pmol/injection) of each oxFA standard were used. For intra-assay precision, the samples were measured three times each on the same day. For interassay precision, the samples were measured on five separate days. Precision values are expressed as the percent value of the standard deviation compared to the mean value (Table 2). Accuracy was determined by measuring three different, known concentrations of oxFA standard five times. Accuracy values are expressed as the percent value of the calculated concentration compared to the known concentration (Table 2).

### 3T3-L1 cell culture and treatment

3T3-L1 preadipocytes were cultured and differentiated as previously described^37,38^. Cells were treated to induce lipolysis on day 10 post-differentiation. Before each experiment, cells were washed with sterile phosphate-buffered saline before undergoing a 1 h serum starve in 3 mL of phenol red-free, high glucose DMEM containing 0.5% fatty acid-free bovine serum albumin. After 1 h, cells were treated with 10 µM isoproterenol, and media was collected after 0, 15, and 45 min. Induction of lipolysis was confirmed by measuring glycerol using a Sigma kit (Hampton, NH) and non-esterified FA using a Fujifilm Wako Diagnostics kit (Richmond, VA) following the included protocols.

### Serum collection

All mice were bred and housed according to the University of Virginia Animal Care and Use Committee (ACUC) requirements and experimentally treated according to ACUC approved protocols. Mice were kept on 12 h light-12 h dark cycle and had ad libitum access to food (normal chow) and water. Twelve 6-month-old C57BL/6J mice (both male and female) were used for basal oxFA measurements. Mice were fasted for 2 h in the morning (fed state) while maintaining free access to water. In order to collect blood, mice were sacrificed by cervical dislocation under isoflurane anesthesia and a cardiac puncture performed. Blood was kept on ice for 20 min to allow for coagulation. The clot was pelleted by centrifugation (1,500 × g, 10 min, 4 °C). The supernatant serum was removed into clean tubes for immediate storage at −80 °C.

### Sample preparation

Lipids were extracted from cellular media or mouse serum spiked with 25 nmol of internal standard (heptadecanoic acid) using a chloroform/methanol extraction. To each glass tube of media or serum, 3 mL of extraction solvent (2:1 chloroform:methanol containing 50 μg/mL BHT to deter further lipid oxidation) was added. The tubes were then vortexed and placed on ice for 30 min before partitioning by centrifugation (2,000 × *g*, 5 min, 4 °C). After centrifugation, the lower organic layer was collected in a separate glass test tube. The remaining aqueous layer was subjected to a second extraction with 1.5 mL of extraction solvent. After vortexing, the samples were again partitioned by centrifugation and the lower organic layer collected and combined with that from the first extraction. The solvent from the collected extractions was evaporated under a stream of nitrogen before being suspended in 100 μL of methanol and analyzed by HPLC-MS/MS.

## Results and Discussion

### Species-dependent condition determination using synthetic standards

**Figure 1:**
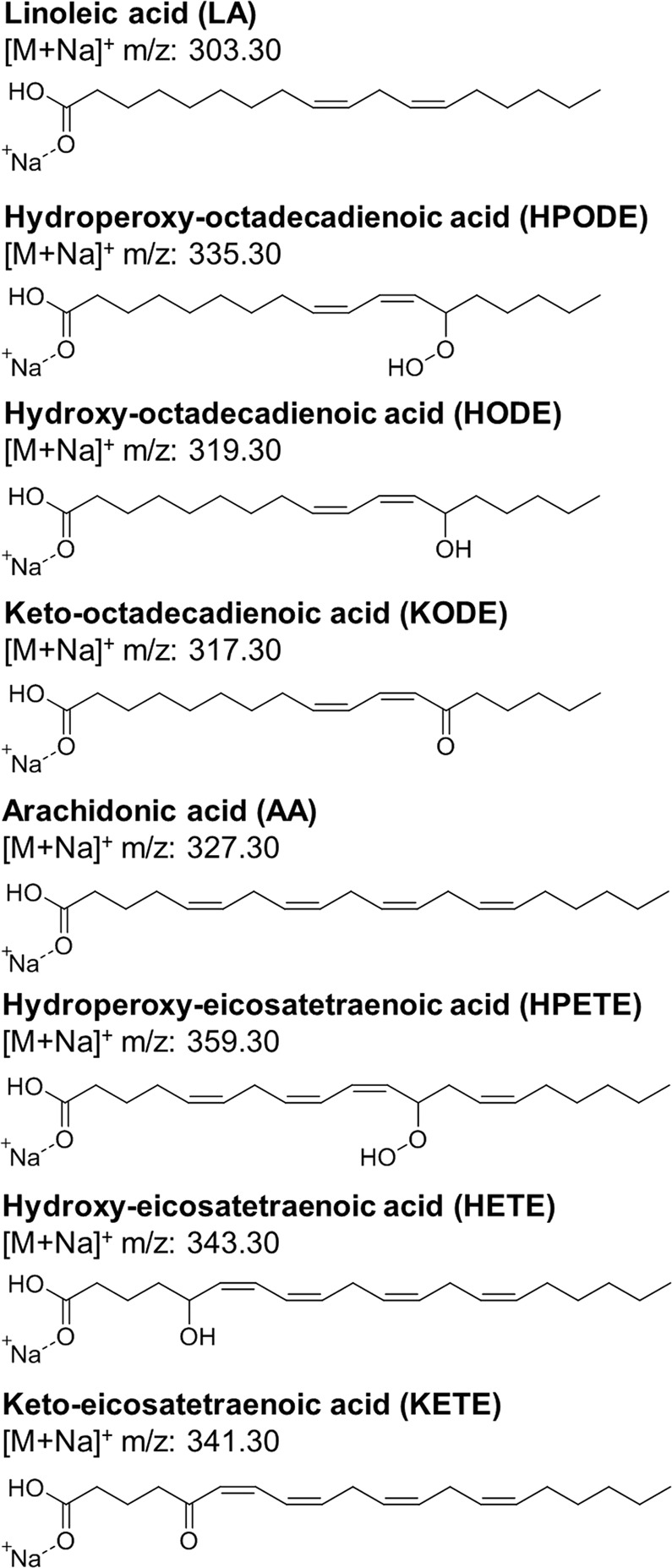
Chemical structures for the sodium adducts of LA and AA as well as their full-length oxidation products (hydroperoxide, hydroxide and ketone).

In addition to allowing measurement with the more compatible positive-ion ionization, previous studies have noted that sodium and other metal adducts result in increased charge-remote fragmentation of lipid species which generates structurally informative product ions^9,22,23,39–47^. This observation has been further extended to oxidized lipid species and has been particularly successful for studying lipid hydroperoxides^9,22,23^. Of note, Ito *et al*. demonstrated the use of alkali metal adducts, particularly sodium, as a fragmentation enhancer of FA hydroperoxides^22,23^. Despite this success, it has yet to be investigated whether these improvements in fragmentation can be extended to other full-length oxFA, such as hydroxides and ketones. To address this, we fragmented and analyzed sodium adducts of full-length oxidation products of the polyunsaturated FA, LA, and AA (Fig. 1).

**Figure 2:**
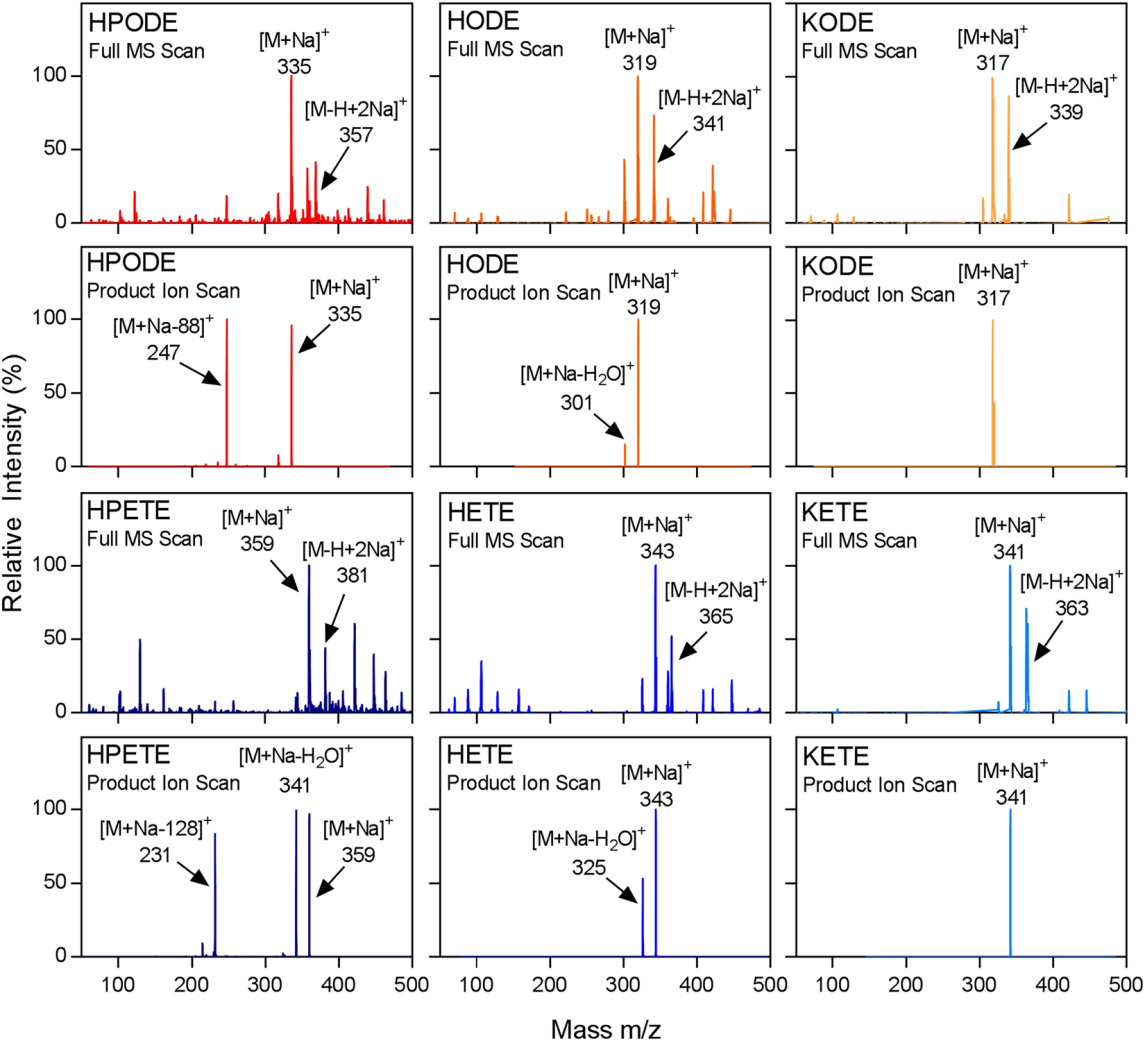
Direct infusion-MS/MS detection and fragmentation of oxFA sodium adducts with positive-ion ionization. Full MS scan of individual oxFA standards in the presence of sodium ions. Product ion scan of oxFA sodium adducts. Peak height is displayed relative to the intensity of the largest peak in the spectra.

The formation and detection of sodium adducts was confirmed by directly infusing individual standards in the presence of sodium ions into the ESI-MS/MS system. Because the inclusion of nonvolatile salts can impair solvent vaporization and lead to instrument buildup, the level of sodium acetate was adjusted to minimize ion suppression and stress on the ESI-MS/MS system while still promoting adequate adduct formation. To further improve solvent vaporization, an elevated interface temperature was chosen that had previously been demonstrated to be compatible with measuring sodium adducts of hydroperoxide oxFA, the least stable of the full-length oxFA species of interest^22,23^. Based on the masses observed in the full-scan spectra, the sodium adduct was formed by coordination of a sodium ion with the carbonyl oxygen in the carboxylic acid of the FA. A less abundant double adduct formed by the displacement of the acidic hydrogen by a second sodium ion was also observed. MS/MS conditions were optimized for enhanced detection of the single adduct of each species (Table 1). As such, the predominant peak in the full MS scan for the oxFA of interest was the single adduct, with the double adduct detectable but at a lower intensity (Fig. 2). Some unidentified, lower intensity peaks were also observed in the full MS scan, particularly of the hydroxide and hydroperoxide species. These peaks are likely due to low abundance contaminants and adducts from the methanol, which has been shown to contain trace levels of ammonium, sodium, and potassium that vary between lots and brands^48,49^. As their presence has been shown to have no adverse effects on calibration and a linear concentration response, no impairment of method capabilities is expected. The formation of the single sodium adduct was also detected for the unoxidized FA (Supplementary Fig. S3).

Fragmentation of the oxFA sodium adducts was examined by product ion scans of the directly-infused standards. As expected based on previous literature, sodium adducts of both hydroperoxides produced stable, characteristic fragment ions (Fig. 2)^22^. 13-HPODE exhibited a fragment of 247 Da, with the loss of 88 Da likely due to cleavage of the bond between C-13 and C-14 and elimination of a hydroxide. 12-HPETE exhibited a fragment of 231 Da with a similar fragmentation pattern (cleavage between C-12 and C-13 and loss of a hydroxide). Despite the enhanced fragmentation of hydroperoxide sodium adducts, the same improvements were not observed for the hydroxides. As seen with many previously published methods, the product ion spectra of HODE and HETE were dominated by the unfragmented molecular ion (in this case, the single sodium adduct) and an uncharacteristic fragment resulting from the loss of water (the most stable product ion formed)^7,9,16,20,22,26,41,42,46,47,50,51^. Due to this stability, the uncharacteristic loss of water was also observed in the full MS scans of the hydroperoxide and hydroxide species (Fig. 2). For the ketone product ion spectra, again, no stable fragments were produced for either KODE or KETE. Similar to their hydroxide products, LA and AA sodium adducts did not display stable fragmentation, as seen by product ion spectra dominated by the molecular ion (Supplementary Fig. S3). Taken together, this data suggests that the enhancement of fragmentation seen by sodium adduct formation for hydroperoxides cannot be generalized to all full-length oxFA species.

### Differential energy qualifier ion monitoring of full-length OxFA

**Figure 3:**
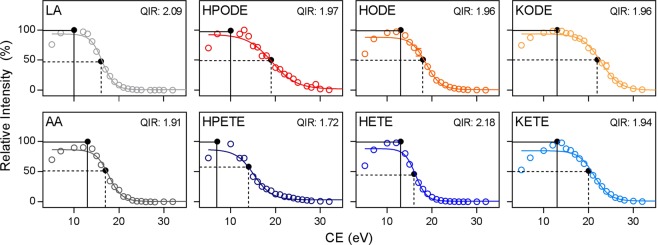
Differential energy transition intensity curves for oxFA and unoxidized FA species. The curves were fit with a sigmoidal non-linear regression. The intensities are displayed relative to the highest intensity transition. The collision energy (CE) yielding the highest intensity transition was designated as the quantitative transition (solid line). The CE yielding an intensity of roughly 50% of maximal was designated at the qualitative transition (dashed line). The qualifier ion ratio (QIR) was calculated based on the intensities of these two transitions. Differential energy curves were performed in triplicate with data expressed as mean ± S.E.M.

Due to the lack of regioisomer-independent characteristic fragments necessary for traditional identification by MS/MS, DiffE QIM was employed. Traditional Qualifier Ion Monitoring (QIM) allows high selectivity by monitoring two or more transitions for each analyte. The most intense transition is used for quantification, while lower intensity transitions are used for identity confirmation. This confirmation is accomplished by calculating the relative intensities between the quantitative and qualitative transitions, known as the QIR^31^. The QIR is a constant property of a species under a given set of MS/MS conditions with adequate chromatographic separation, regardless of the matrix^32,34,52^. Due to the lack of regioisomer-independent, stable fragmentation of oxFA, traditional QIM is not possible. However, Hellmuth *et al*. recently established the technique of DiffE QIM for use with FA, which relies on the unique relationship of collision energy (CE) to molecular ion signal intensity^30^. The quantitative and qualitative transitions are set to the same pseudo-molecular mass transition with the parent ion and product ion mass set to the molecular ion adduct but with increased CE for the qualitative transition (Table 1). The ratio of these transitions was demonstrated to specifically identify FA in biological samples^30^. As such, we looked to extend this technique for the first time to oxFA species. The unique relationship between the intensity of the pseudo-molecular transition and CE was determined for the unoxidized FA and their full-length oxFA products (Fig. 3). The CE that yielded the highest transition intensity was designated for the quantitative transition, while the CE that yielded an intensity of roughly 50% was chosen for the qualitative transition. Based on these differential energy transitions, the QIR was determined for each oxFA (Table 1). Based on industry and government standards, with a qualitative transition intensity of 50% of the quantitative transition, the acceptable QIR range for identification is 75–125% of the QIR of the reference standard^30–36^.

### Chromatographic separation and quantification of synthetic standards

Recent studies examining oxFA have been focused on regioisomer identification and have thus relied heavily on chromatographic separation of these species by chiral columns^22,23^. In contrast, our goal was to quantitate full-length oxFA independent of regioisomer, since it is probable that the sum total of oxidation products, rather than the formation of a specific regioisomer, is more relevant in the pathophysiology of chronic oxidative stress diseases^12,28^. As such, the chromatographic method employed should separate the oxFA from other potentially conflicting lipid entities while still allowing collective elution of all the regioisomers of a given oxFA species in a single peak. To this end, reverse phase chromatography was selected, since the primary distinguishing characteristic of oxFA compared to other lipids is their increased polarity imparted by the oxidized moiety and amplified by their relatively small size. As previously mentioned, if not properly accommodated, the inclusion of nonvolatile salts, such as sodium acetate, can reduce performance of the ESI-MS/MS system by leading to residual salt within the system. This build-up could further confound our results by increasing the formation of the double sodium adduct of the oxFA. To limit these potential issues, sodium acetate was confined to the primary elution solvent (Solvent B), and a 10 column-volume wash with salt-free Solvent A was programmed between samples. The inclusion of a thorough wash prevented the accumulation of salt within the HPLC and ESI and allowed for the stable formation of the single sodium adduct. This can be demonstrated by the precision and accuracy values calculated during validation of the method (Table 2). The washes had the added benefit of reducing carryover between samples, a common problem in lipid analysis^53^. To maximize solvent vaporization and thus analyte detection, a low flow rate, which gave the highest signal intensity for the standards, was selected.

**Figure 4:**
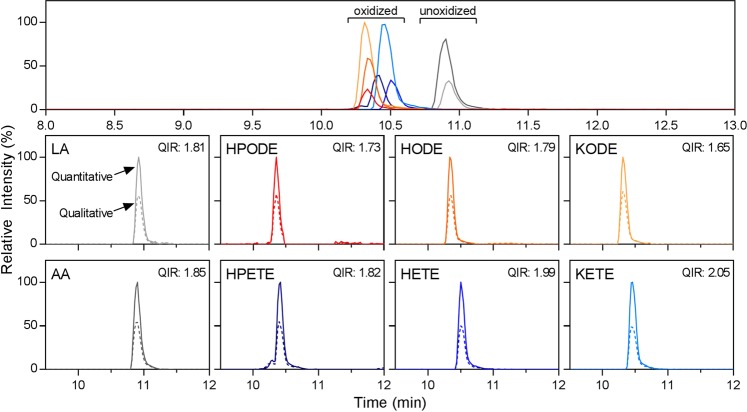
HPLC-MS/MS approach to quantification of oxFA sodium adducts with positive-ion ionization. Chromatogram of a 10 μM standard mix of oxFA species. Zoomed chromatograms of the individual analytes with both the quantitative (solid line) and qualitative transitions (dashed line) visualized with the calculated qualifier ion ratio (QIR). Peak height is displayed relative to the highest intensity peak in each chromatogram.

Under these chromatographic conditions, high-quality peaks with extremely stable retention times were obtained with a distinct separation of the oxFA standards from their unoxidized counterpart (Table 2, Fig. 4). The oxidized species were found to elute earlier, reflective of their increased polarity. Furthermore, peaks for the quantitative and qualitative transitions were observed to have corresponding retention times and fell in the expected range of QIR for each species (Fig. 4). Though there was less than a minute separation between the oxidized and unoxidized species, this was deemed sufficient separation in order to maintain a short overall runtime. In fact, the method was able to be even further reduced without significant effects on the method capability, yielding a rapid 11 min runtime (Supplementary Fig. S2). If increased separation is the priority, the published HPLC method can easily be adapted for use with a longer column (Supplementary Fig. S2).

Linearity over a 5 log_10_ range was achieved for each analyte of interest, with a coefficient of determination (R^2^ value) of at least 0.988 (Supplementary Fig. S1). As shown in the full MS scans of oxFA, the double adduct is present in all samples in addition to the single adduct of interest (Fig. 1). To ensure that the presence of the double adduct does not hinder reliability, the precision and accuracy of the method were analyzed. The intra-assay and interassay precision of the analytes were in the range of 0.8–6.5% and 4.3–10.8%, respectively (Table 2). Based on comparison to a calibration curve of the pure reference standard, the accuracy of samples with a known concentration were all within the accepted range of 80–120% (Table 2). As a result, we concluded that presence of the double adduct was sufficiently accounted for in our method design. The LOQ for each species was determined to be the concentration at which the qualitative transition has a signal-to-noise ratio of greater than 3:1 (Table 2). Because this method relies on DiffE QIR, the LOD and LOQ are identical, as a species cannot be definitively identified in the absence of the qualitative transition. The LOQ for the oxFA species were determined to be 0.1 pmol. The LOQ for the unoxidized FA species were lower at 0.01 pmol, likely due to their increased stability compared to oxFA. These LOQ values are similar to reported LOQ values for unoxidized FA quantification by HPLC-MS/MS using the QIR approach to species confirmation^30^. The LOQ values for our oxFA and previously published unoxidized FA methods are slightly above those reported for HPLC-MS/MS methods quantifying derivatized FA^54,55^.

### Regioisomer-independent HPLC-MS/MS analysis of complex oxidized mixtures

**Figure 5:**
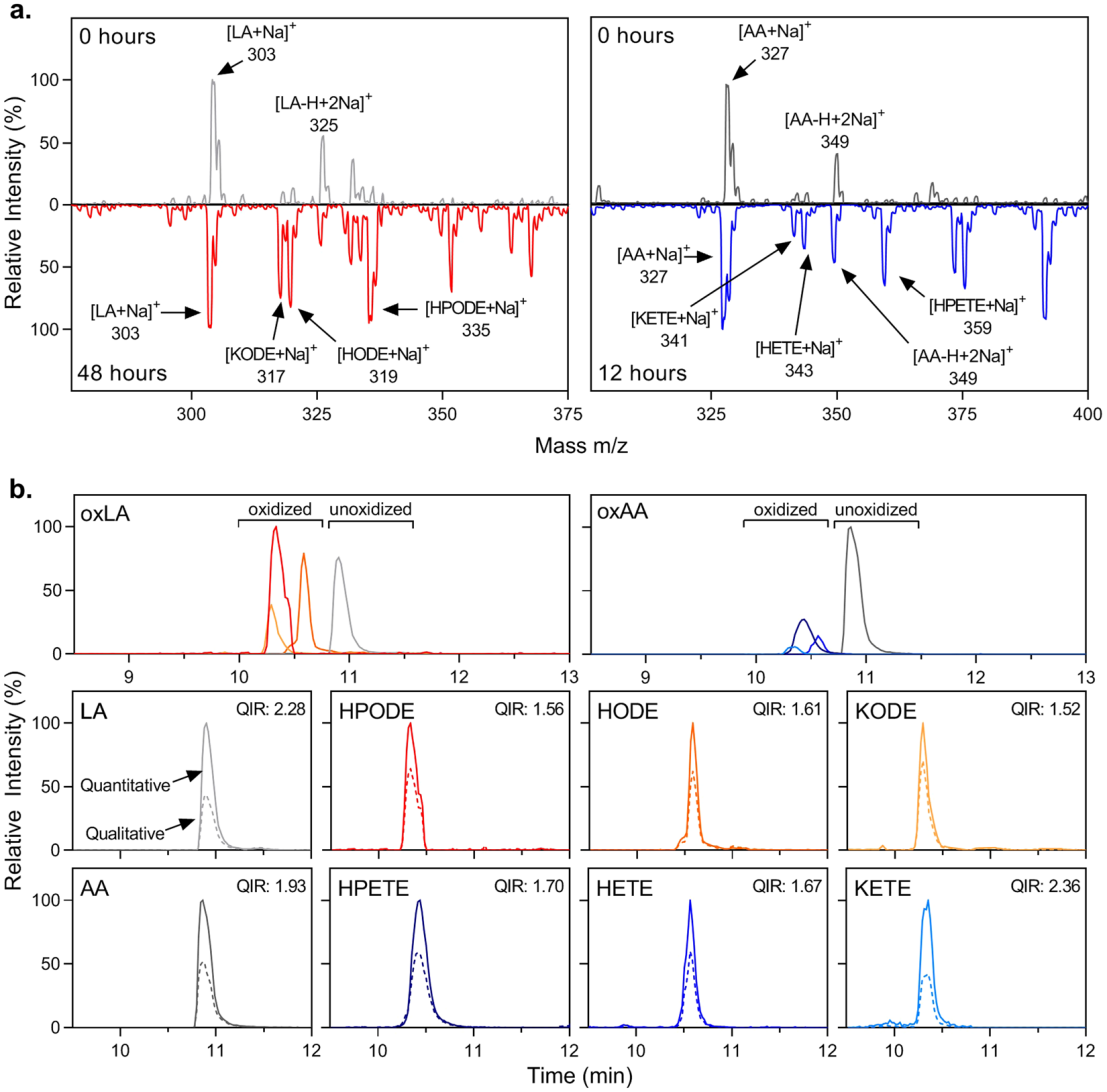
Method validation with a complex mixture of species and regioisomers. (**a**) Full MS scan of oxFA mixtures produced by auto-oxidation of LA and AA for specified time period. Both unoxidized and auto-oxidized spectra were measured under the optimized conditions for the unoxidized species. Peak height is displayed relative to the highest intensity peak in the spectra. (**b**) Chromatograms of 100 μM auto-oxidized LA and AA for specified amount of time. Zoomed individual analyte chromatograms depict both the quantitative (solid line) and qualitative transitions (dashed line). Peak height is displayed relative to the highest intensity peak in each chromatogram.

Because characterization and optimization were performed with pure standards of a single regioisomer, a mixture of oxFA was required to ensure the optimized conditions could definitively identify the full-length species of interest within a mixture, independent of regioisomer. To create a diverse mixture of species and regioisomers, auto-oxidation of LA and AA was performed. Progression of oxidation was monitored over time by directly infusing the mixture in the presence of sodium ions into the ESI-MS/MS, and the time point with the highest intensity of full-length species is represented (Fig. 5a). This occurred much more rapidly for AA than LA (12 h compared to 48 h), likely due to the increased number of susceptible allylic carbons. At each of these time points, a significant peak corresponding to the unoxidized FA was still observed. Based on comparison with the full-scan and product ion mass spectra of the pure standards, the presence of sodium adducts of the hydroperoxide, hydroxide, and ketone oxidation products for both LA and AA could be identified, indicating that the determined species-specific conditions could be used to detect the full-length oxidized species of interest, regardless of regioisomer, within a complex lipid mixture.

To ensure that all regioisomers eluted collectively under the determined chromatographic conditions, the auto-oxidized mixture containing a variety of oxidized species and regioisomers was again employed. Similar to the chromatogram observed for the standards, separation of the full-length oxFA from their unoxidized counterparts was obtained, with a singular peak for each analyte observed, indicating unified elution of all regioisomers (Fig. 5b). Furthermore, species identity within the complex mixture was confirmed, since the QIR for each analyte fell within the acceptable range. To confirm our ability to quantitate total levels of full-length oxFA, various time points throughout auto-oxidation of LA and AA were collected, and the production of each full-length oxFA and reduction of its unoxidized counterpart were quantified over time. In agreement with the observation by direct infusion analysis, AA oxidized on a more rapid timescale than LA (Supplementary Fig. S4). Of the time points analyzed, peak levels of full-length oxFA were achieved after 48 h for LA and 12 h for AA. The percentage of full-length oxFA present reached a higher maximum for LA compared to AA. This is likely due to the different rates of oxidation progression for LA and AA. Just as AA forms full-length oxFA more quickly due to the increased number of susceptible allylic carbons, these species undergo further oxidation at a more rapid pace. As such, equilibrium of full-length oxFA formation and further oxidation is likely established at a lower level for AA than LA. Based on this data, our HPLC-ESI-MS/MS method implementing DiffE QIM was validated for measuring and quantifying full-length oxFA species independent of regioisomer within a complex mixture.

### Quantification of full-length OxFA species in biological samples

In order to confirm our ability to measure these lipid species in a regioisomer-independent manner within a complex biological sample, we next examined the basal levels of oxFAs circulating in mouse serum. Before HPLC-ESI-MS/MS analysis, the serum was subjected to a simple chloroform/methanol lipid extraction. While acidified extraction methods are frequently employed for more polar lipids, this was found to not be compatible with full-length oxFA species due to their acid lability^20^. To correct for extraction efficiency, heptadecanoic acid (C17:0) was implemented as an internal standard. C17:0 has similar polarity and partitioning qualities as LA and AA but is not naturally occurring within mammalian cells, making it a viable internal standard for our purposes^56^. The MS and HPLC conditions for the C17:0 sodium adduct were determined following the same procedure as for the oxFA species (Supplementary Fig. S6).

**Figure 6:**
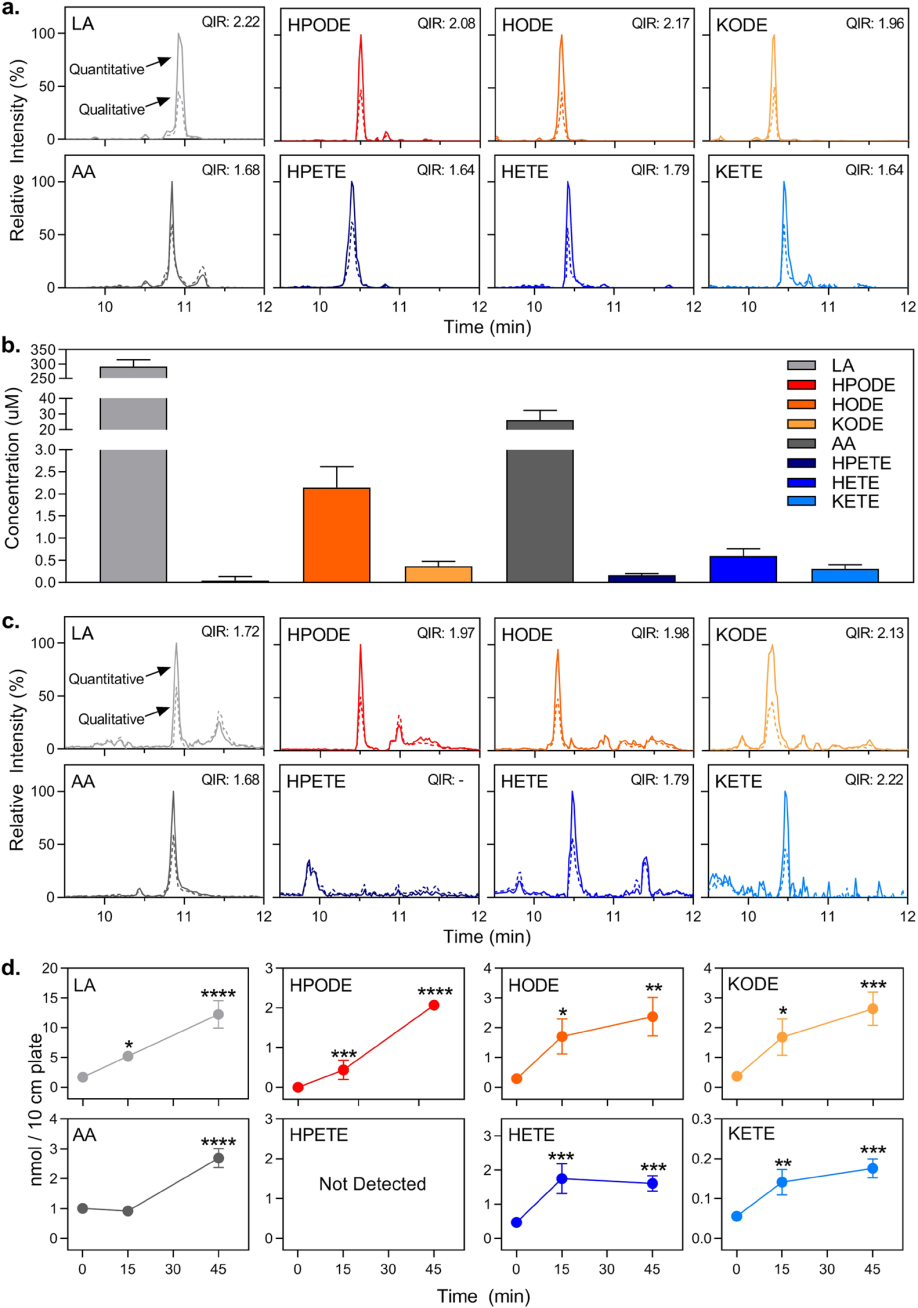
Quantification of oxFA species in biological samples. (**a**) Representative chromatogram of oxFA in basal mouse serum samples. Quantitative (solid line) and qualitative transitions (dashed line) visualized with the calculated QIR. Peak height is displayed relative to the highest intensity peak in each chromatogram. (**b**) Concentration of oxFA in mouse serum. Blood was collected by cardiac puncture from anesthetized mice. Serum was isolated, subjected to a lipid extraction, and measured by HPLC-MS/MS. Data shown is from 12 mice and expressed as mean ± S.E.M. (**c**) Representative chromatogram of oxFA in 3T3-L1 cell media samples. Quantitative (solid line) and qualitative transitions (dashed line) visualized with the calculated QIR. Peak height is displayed relative to the highest intensity peak in each chromatogram. (**d**) Levels of oxFA released from 3T3-L1 adipocytes into cellular media during lipolysis. 3T3-L1 adipocytes were serum starved in high glucose DMEM + 0.5% FA-free BSA for 1 h before being treated with 10 μM isoproterenol to stimulate lipolysis. Media was collected after 0, 15, and 45 min of treatment, subjected to a lipid extraction, and measured by HPLC-MS/MS. Cellular treatments and analysis were performed in quadruplicate with data expressed as mean ± S.E.M. Asterisks indicate significant difference compared to 0 min (*p < 0.05, **p < 0.01, ***p < 0.001, ****p < 0.0001, respectively).

Based on comparison of peak retention time and QIR to the oxFA standards, all FA and oxFA species were identified (Fig. 6a). The levels of circulating LA and AA were found to be 291 µM and 26 µM, respectively, matching previously measured concentrations in the literature (Fig. 6b)^57–61^. The concentrations of LA-derived HPODE, HODE, and KODE were found to be 45 nM, 2.2 µM, and 0.4 µM, respectively. HODE had the highest concentration, likely because it is the most stable of the three species. The concentrations of AA-derived HPETE, HETE, and KETE were found to be 161 nM, 600 nM, and 306 nM, respectively. Again, the hydroxyl product, HETE, was measured at the highest levels due to its increased stability. The sum of just these full-length oxidation products were found to compose approximately 1% of circulating LA, whereas they makeup approximately 4% of circulating AA. These results correspond with our auto-oxidized time course where we demonstrated that AA oxidizes nearly four times as quickly as LA (Fig. 5 and Supplementary Fig. S4).

We have previously shown that extracellular full-length oxFA are capable of inducing a proinflammatory macrophage phenotype^12^. Furthermore, it has long been reported that adipocytes exhibit elevated basal levels of lipolysis in obesity^62^. As such, we decided to further validate our method by investigating whether full-length oxFA are released during lipolysis and quantifying their production. For this purpose, we utilized the 3T3-L1 mouse cell line as an *in vitro* model of adipocytes. Differentiated 3T3-L1 adipocytes were treated with isoproterenol, an exogenous catecholamine that stimulates β-adrenergic receptors leading to the potent induction of lipolysis^38,63^. The cellular media was collected 0, 15, and 45 min after treatment, and lipolysis was confirmed by measuring the release of the products non-esterified FA (NEFA) and glycerol (Supplementary Fig. S5). All the oxFA species of interest were found to be present in the cellular media after induction of lipolysis except for HPETE, again based on peak retention time and QIR (Fig. 6c). The lack of HPETE in our cultured cell sample is likely due to its high reactivity and cellular peroxidase-driven breakdown to HETE^64^. Already by 15 min, our method was able to quantify significant levels of the detected oxFA species. These levels were maintained or continued to increase as quantified at 45 min, indicating that oxFA are released from adipocytes during lipolysis and can be measured using our method (Fig. 6d). Based on these results, our method is capable of measuring full-length oxFA species in an isoform-independent manner in both cellular media and serum and can likely be extended to other biological samples.

## Conclusion

Despite the importance of oxFA as signaling molecules and their roles in the pathophysiology of metabolic disease, the development of HPLC-ESI-MS/MS methods to quantify these species has been slow. Recent methods developed to measure FA oxidation have focused heavily on identifying specific regioisomers either through an untargeted approach with only relative quantitation capability or through a targeted approach, of which some methods allow absolute quantitation^7,16,20–25^. However, it is likely that the sum total oxidation products, rather than the formation of one particular regioisomer, is more important in pathologies implicating chronic oxidative stress^12,28^. Unfortunately, methods focused on measuring these species irrespective of regioisomer rely predominantly on HPLC with chemiluminescence detection, which, while easily quantitated, is inherently limited in its specificity^8,26,27^. These methods usually measure oxFA solely based on the oxidized moiety and are unable to distinguish between individual FA backbones^8,26,27^. Therefore, a method that could quantitate specific oxFA in a regioisomer-independent manner would be highly biologically relevant. Our method represents significant progress toward this goal.

While it is true that untargeted methods allow for a far greater number of species to be monitored, these methods usually rely on relative quantitation, are amenable only to measurement of specific regioisomers, and are generally less facile to implement than targeted methods. Our targeted method outlined in this report is able to identify and absolutely quantify multiple full-length oxFA species independent of regioisomer with a relatively short runtime. We have shown our method is highly sensitive, without requiring time-consuming and destructive derivitization steps, due to the utilization of sodium adducts to take advantage of positive-ion ionization and DiffE QIM to quantify based on regioisomer-independent, high intensity, pseudo-molecular transitions. The implementation of DiffE QIM also circumvents the difficulty of fragmentation since it does not rely on characteristic fragmentation. To our knowledge, this is the first study implementing DiffE QIM to measure oxFA, and based on our success measuring full-length oxFA, could likely be applied to other oxFA species for regioisomer-independent quantification. Furthermore, our results in cellular media and mouse serum indicate that our approach could be extended for oxFA quantification in other biological samples.

Additionally, as lipid oxidation frequently occurs on esterified fatty acids, this method could be readily applied to measuring the oxidized acyl chains of triglycerides and phospholipids with the addition of a lipase treatment to the sample preparation. This is of particular importance as we have previously shown that oxidized arachidonic acid-containing phosphotidylcholine drives pro-inflammatory and antioxidant gene expression in macrophages, and these effects can be recapitulated with products of nonesterified arachidonic acid oxidation^13^. Numerous studies have identified distinct biological functions not only for a variety of individual oxidized lipid species, but also for sets of oxidized lipids with similar functional moieties. Even so, prior to this study, there has not been a regioisomer-independent, targeted HPLC-ESI-MS/MS method with which to measure oxFA species. Our HPLC-ESI-MS/MS method provides a simple and reliable means for measuring oxFA species and elucidating their growing role in human disease. The ability to faithfully quantify individual oxFA species, regardless of regioisomer, is a currently unmet need in the oxidized lipids field, and should be further pursued in order to measure oxFAs in a robust and comprehensive manner in biological samples.

## Supplementary information

Supplementary Data

## Data Availability

The datasets generated during and/or analyzed during the current study are available from the corresponding author upon reasonable request.
