# Supplementary material for: Regioisomer-independent quantification of fatty acid oxidation products by HPLC-ESI-MS/MS analysis of sodium adducts
Source: Sci Rep. 2019 Aug 1;9:11197. doi: 10.1038/s41598-019-47693-5 (PMC6671977; doi:10.1038/s41598-019-47693-5)
Supplement: Supplementary file 1 — Supplementary Data [file 41598_2019_47693_MOESM1_ESM.pdf]

SUPPLEMENTARY DATA

Regioisomer-Independent Quantification of Fatty Acid Oxidation Products by HPLC-ESI-MS/MS Analysis of Sodium Adducts

Katelyn W. Ahern, Vlad Serbulea, Catherine L. Wingrove, Zachary T. Palas, Norbert Leitinger, Thurl E. Harris*

Department of Pharmacology, University of Virginia Health System, Charlottesville, VA 22908, United States

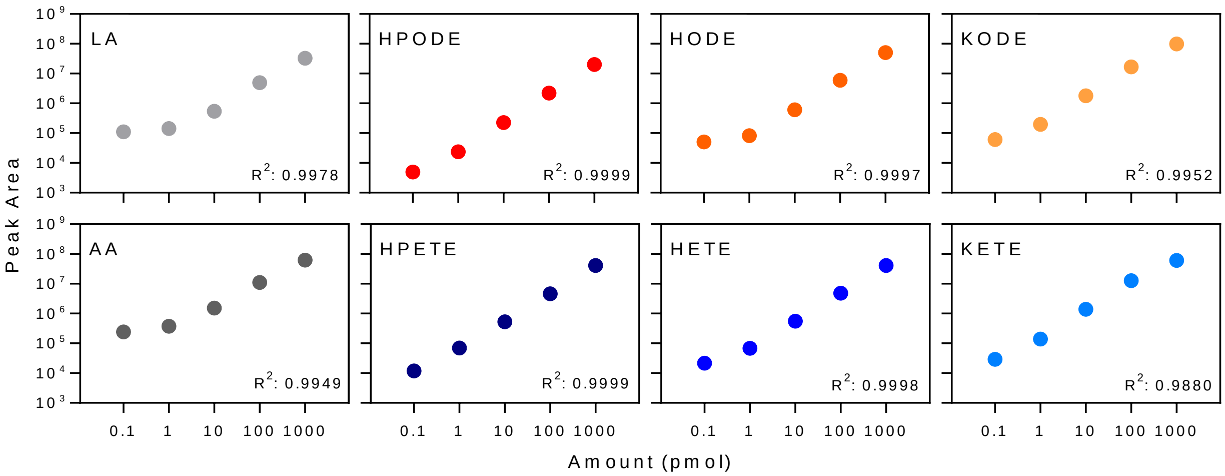

Supplementary Figure S1. HPLC-MS/MS calibration curves for oxFA and unoxidized FA species. Linearity was achieved over a 5 $\log_{10}$ range for each analyte with a high coefficient of determination (R^2 value). Quantification of oxFA in samples was based on comparison to the corresponding curve. Calibration curves were performed in triplicate over the course of a 22 h constant run with data expressed as mean ± S.E.M.

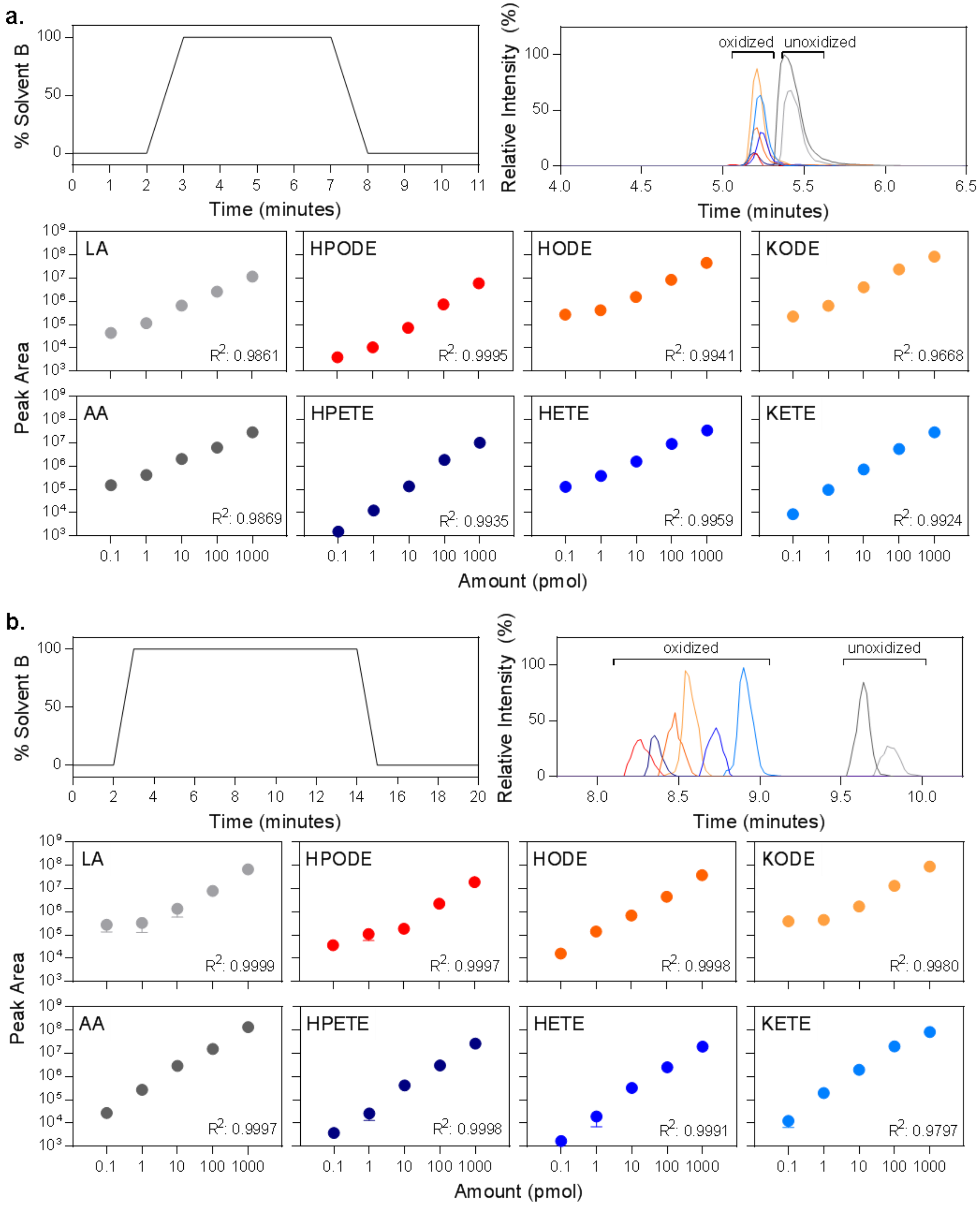

Supplementary Figure S2. Alternate, compatible HPLC methods. **a.** Chromatogram and calibration curves generated using a shorter runtime HPLC method **b.** Chromatogram and calibration curves using a HPLC method with a longer column. Calibration curves were performed in triplicate with data expressed as mean ± S.E.M.

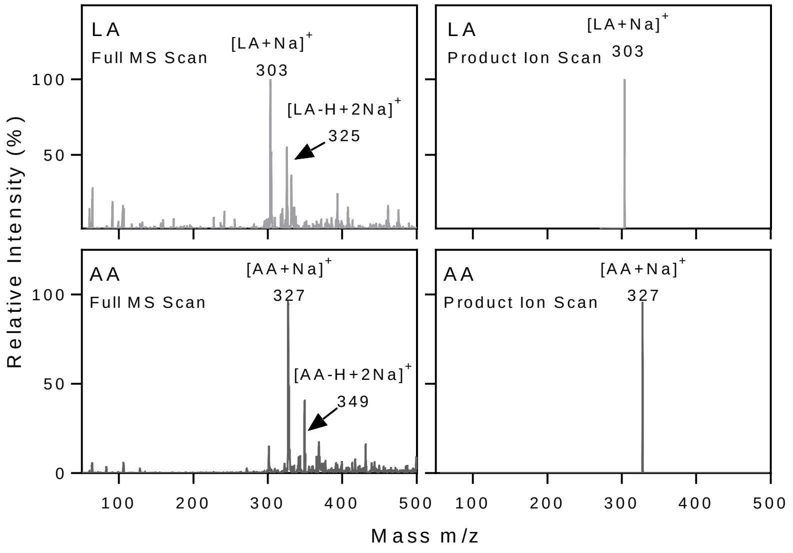

Supplementary Figure S3. Direct infusion-MS/MS detection and fragmentation of unoxidized LA and AA sodium adducts with positive-ion ionization. Full MS scan of individual LA and AA standards in the presence of sodium ions. Product ion scan of LA and AA sodium adducts. Peak height is displayed relative to the intensity of the largest peak in the spectra.

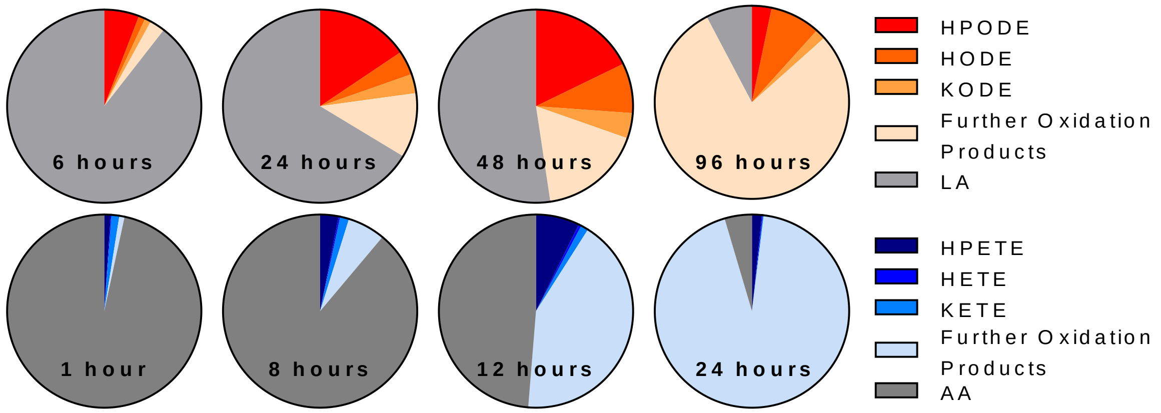

Supplementary Figure S4. Method quantification validation with a complex mixture of auto-oxidized species and regioisomers. The auto-oxidation of LA over 96 h and AA over 24 h, as represented by pie charts visualizing the relative amount of each species. Further oxidation products were defined as the portion of the known starting amount of unoxidized FA that could not be accounted for by the quantification of our species of interest. Auto-oxidation time course was performed in triplicate.

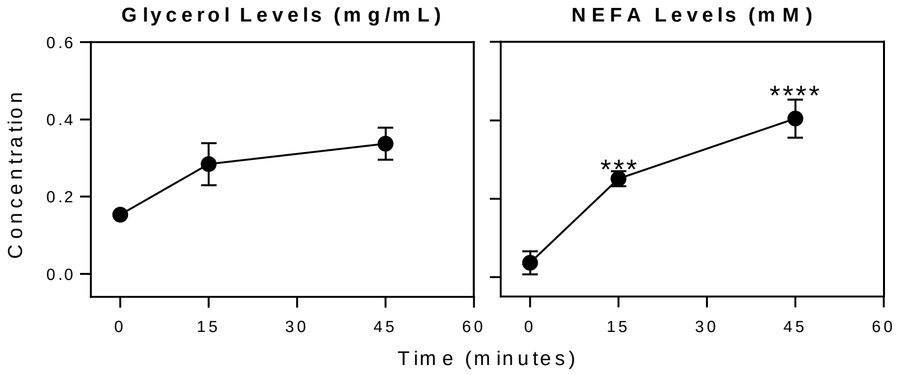

Supplementary Figure S5. Induction of lipolysis in 3T3-L1 adipocytes. 3T3-L1 adipocytes were serum starved in high glucose DMEM + 0.5% FA-free BSA for 1 h before being treated with 10 μM isoproterenol to stimulate lipolysis. Media was collected after 0, 15, and 45 min of treatment. Glycerol and NEFA, the products of lipolysis, were measured in the media using their respective kits to confirm induction. Cellular treatments and analysis were performed in quadruplicate with data expressed as mean ± S.E.M. Asterisks indicate significant difference compared to 0 min (*** $p<0.001$, **** $p<0.0001$, respectively).

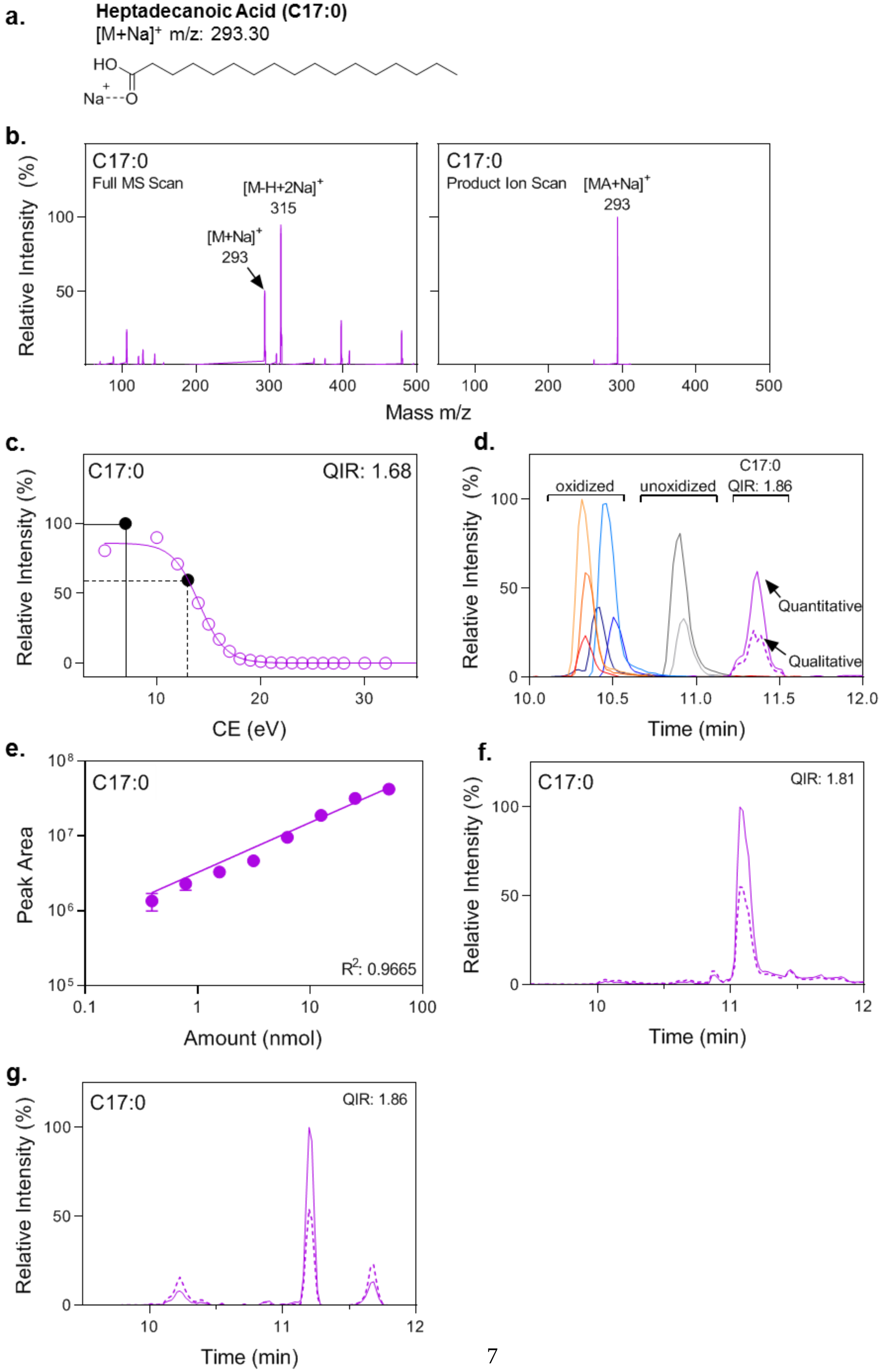
a.
Heptadecanoic Acid (C17:0)
[M+Na]+ m/z: 293.30
HO
Na
O
b.
C17:0
Full MS Scan
[M-H+2Na]+
315
[M+Na]+
293
C17:0
Product Ion Scan
[MA+Na]+
293
Relative Intensity (%)
Mass m/z
c.
C17:0
QIR: 1.68
Relative Intensity (%)
CE (eV)
d.
oxidized
unoxidized
C17:0
QIR: 1.86
Quantitative
Qualitative
Relative Intensity (%)
Time (min)
e.
C17:0
R2: 0.9665
Peak Area
Amount (nmol)
f.
C17:0
QIR: 1.81
Relative Intensity (%)
Time (min)
g.
C17:0
QIR: 1.86
Relative Intensity (%)
Time (min)

Supplemental Figure S6. Internal standard conditions and validation. **a.** Chemical structure for the sodium adduct of C17:0. **b.** Direct infusion-MS/MS full MS scan of C17:0 standard in the presence of sodium ions. Product ion scan of C17:0 sodium adducts. Peak height is displayed relative to the intensity of the largest peak in the spectra. **c.** The differential energy transition intensity curve fit with a sigmoidal non-linear regression. The intensities are displayed relative to the highest intensity transition. The collision energy (CE) yielding the highest intensity transition was designated as the quantitative transition (solid line). The CE yielding an intensity of roughly 50% of maximal was designated at the qualitative transition (dashed line). The differential energy curve were performed in triplicate with data expressed as mean ± S.E.M. **d.** HPLC-MS/MS chromatogram of 10 μM standard mix of oxFA species. C17:0 quantitative (solid line) and qualitative transitions (dashed line) visualized with the calculated QIR. Peak height is displayed relative to the highest intensity peak in chromatogram. **e.** HPLC-MS/MS calibration curve for C17:0. Linearity was achieved over a 5 $\log_{10}$ range with a high coefficient of determination (R^2 value). Quantification in samples was based on comparison to the curve. Calibration curve was performed in triplicate with data expressed as mean ± S.E.M.. **f.** Representative chromatogram of C17:0 in 3T3-L1 cell media samples. C17:0 quantitative (solid line) and qualitative transitions (dashed line) visualized with the calculated QIR. Peak height is displayed relative to the highest intensity peak in chromatogram. **g.** Representative chromatogram of C17:0 in basal mouse serum samples. C17:0 quantitative (solid line) and qualitative transitions (dashed line) visualized with the calculated QIR. Peak height is displayed relative to the highest intensity peak in chromatogram.
